# Underwater wireless sensor network-based multihop data transmission using hybrid cat cheetah optimization algorithm

**DOI:** 10.1038/s41598-023-37952-x

**Published:** 2023-07-04

**Authors:** M. M. Vijay, J. Sunil, V. G. Anisha Gnana Vincy, M. IjazKhan, Sherzod Shukhratovich Abdullaev, Sayed M. Eldin, Vediyappan Govindan, Hijaz Ahmad, Sameh Askar

**Affiliations:** 1 SCAD College of Engineering and Technology, Tirunelveli, India; 2 Department of Computer Science and Engineering, Annai Vailankanni College of Engineering, Kanyakumari, India; 3 Department of Computer Science and Engineering, Danish Ahmed College of Engineering, Chennai, India; 4 Department of Mathematics and Statistics, Riphah International University I-14, Islamabad, 44000 Pakistan; 5 Mechanical Engineering, Lebanese American University, Kraytem, Beirut, 1102-2801 Lebanon; 6 Faculty of Chemical Engineering, New Uzbekistan University, Tashkent, Uzbekistan; 7 Department of Science and Innovation, Tashkent State Pedagogical University Named After Nizami, Bunyodkor Street 27, Tashkent, Uzbekistan; 8 Center of Research, Faculty of Engineering, Future University in Egypt, New Cairo, 11835 Egypt; 9 Department of Mathematics, Dmi St John The Baptist University, 800, Lilongwe, Central Africa Malawi; 10 Department of Mathematics, Hindustan Institute of Technology and Science, Rajiv Gandhi Salai (OMR), Padur, Kelambakkam, 603103 Tamil Nadu India; 11 Section of Mathematics, International Telematic University Uninettuno, Corso Vittorio Emanuele II, 39, 00186 Roma, Italy; 12 Department of Statistics and Operations Research, College of Science, King Saud University, P.O. Box 2455, Riyadh, 11451 Saudi Arabia

**Keywords:** Computational science, Computer science

## Abstract

For the conservation and sustainable use of the oceanic environment, monitoring of underwater regions is ineluctable and is effectuated with the aid of an underwater wireless sensor network. It is accoutered with smart equipment, vehicles and sensors and utilized for the transmission of acquired data from the monitoring region and forwarded to the sink nodes (SN) where the data are retrieved. Moreover, data transmission from sensor nodes to SN is complicated by the aquatic environment's inherent complexities. To surpass those issues, the work in this article focusesto propose a Hybrid Cat Cheetah optimization algorithm (HC^2^OA) that purveys the energy efficient clustering based routing. The network is then partitioned into numerous clusters, each of which is led by a cluster head (CH) and comprised of many sub-clusters (CM). Based on the factors such as distance and residual energy the CH selection is optimized and collects data from the respective CMs and forwarded to the SN with a multi-hop transmission approach. The proposed HC^2^OA chooses the optimized multi-hop route from the CH to SN. Thus mitigates the complexities over multi-hop routing and CH selection. Simulations are effectuated in the NS2 simulator and analyzed the performance. The results of the study show that the proposed work has significant advantages over state-of-the-art works in terms of network lifetime, packet delivery ratio, and energy consumption. The energy consumption of the proposed work is 0.2 J with a packet delivery ratio is 95%.The network life time of proposed work, with respect to the coverage area around 14 km is approximately 60 h.

## Introduction

The underwater wireless sensor network^1^ is a platform utilized to measure an enterprise’s ability within a defined location; it is furnished with real-time data collection and automobiles designed to cooperate through communication channels. The earth's crust sink gathers information from edge devices. Echolocation systems^2^ are used in aural perception to describe the shoreline, guide submersibles, and detect below objects. Submerged diagnostics for material assessment, colorimetry, and fluorophotometry for measuring various factors are examples of electro-optic biosensors used in AtlanticAdventures.Currently, the subterranean flow of information is accomplished through several of biophysical factors, including the geomagnetic force, photonic domain, and sound waves^3^. Reverberant listening is important for data transmission because saltwater absorbs electrostatic and photonic waves faster than echoes. The bottom tier of the transceiver level is the intermediate security system framework for low power wide area networks, which primarily distributes numerous basement hubs in a fair and efficient circumstance. This framework is essential for ensuring information sharing. Auditory systems, broadcasting, and free space optical interactions are a few examples of communication technologies^4^ that are used or thought about when communicating underwater. Two significant detectors that are frequently employed for underwater object detection are transducers and cinematography. It can provide information about underwater sceneries even in low- and no-visibility situations sensors can get knowledge about submerged sceneries including in medium and high and no-visibility situations since they are susceptible to spatial crystal structures. Similar to how organisms use echolocation to share information, cordless deep ocean transmission employs water to transponder another audio signal.

When radio waves enter the water, they quickly lose their radio frequency. Ultrasonic signals^5–7^ sent by underwater equipment mostly include off another ground without anyone ever penetrating. However, Wi-Fi waves are completely useless underwater. Constructing wireless routers underwater could be feasible in the long term, but it is not a viable choice right now. Wireless multi-hop networks^8–10^ do not require central power or suppliers to operate because nodes can connect with one another across wireless channels. Nodes can work together by reflecting or transferring one another’s packets, potentially including a large number of secondary intermediate nodes. Single-hop communication^11–13^ lets the material explanation connect directly to the sink nodes, while multi-hop communication requires a transceiver to send supplier data to the sink. Multipath propagation refers to the numerous options and making by transmission as it progresses toward its destination. It’s possible that refraction, dispersion, or perhaps diffraction caused the pathways, these routes involve several hops. In a bid to generate more comprehensible link recommendations, non-linear–linear^14–16^, and no argumentation have recently received a lot of attention. In a bid to generate more comprehensible link recommendations, non-linear- linear, and non-argumentation have recently received a lot of attention. In contrast, there hasn’t been much emphasis on assessing their interpretability, and our findings show that many of the pathways suggested by various frameworks are truly absurd^17–19^. The systems are able to increase connectivity and expand a channel’s communication range. Furthermore, transmission over a number of low links might need less energy and resources than communication over a number of wide ones. In comparison to their sound waves and Baseband equivalents, submarine optical wireless communications may enable larger storage rates at low response levels because of their higher throughput^20–22^.

### Motivation and contribution

The main issue of UWSN is that the sensors get fault easily and it is necessary to maintain the lifetime of the network. Meanwhile, the selection of CH is also critical since the selected CH has less residual energy it will die earlier and impacts the performances of UWSN. The selection of routing is also to be considered to reduce information loss, delay, and throughput. To surmount those issues, we have proposed a novel approach known as HC^2^OA-based UWSN. Some of the contributions are listed below,

- The system model and energy models of the UWSN are explained which is used for further processing.
- The drift concept is added to illustrate the dynamic nature of the system and how often the nodes change their position with the ocean current.
- The proposed method has two phases: CH selection and data transmission. Most CH selection protocols ignore residual energy, which is more important.
- The residual energy and the shortest distance were used to the SN sensor to reduce delay, information loss, and network lifetime.
- This work also allows multi-hop routing with the proposed HC2OA, which optimizes data transmission routing.
- The proposed work can be utilized collaboratively used for the monitoring and gathering of underwater vehicles and data and used interactively between the sensors nodes underwater and ground base stations.

### Problem statement

While deploying the UWSN system practically, the restrictions underwater might have impacted the network performances. The deployment of large-scale multi-hop UWSN depicts more significant impacts than the small-scale network. This brings challenges and packet impairments also occur with the accumulated impacts in the multi-hop data transmission. To surmount these challenges, we proposed or establish an underwater routing mechanism that can be utilized with large-scale networks and thus ensures a higher packet delivery ratio^23–25^. This is the main aim of our work to improve the packet delivery ratio with the minimum utilization of energy by the network.

The remaining sections are structured as follows: In Sect. "Literature survey" examines the works currently considered to be state-of-the-art, pointing out both their strengths and weaknesses. Section "Background" provides the necessary diagram and equations to explain the system model. Section "Proposed clustering routing algorithmic demonstration" explains the method that has been suggested. In the Result sand discussion section, we examine and contrast the results.

## Literature survey

To reduce the device's energy consumption and tackle the problem of submerged source nodes' electricity consumption balancing, Wang et al.^26–28^ presented a dynamic clustering K-means (DC-K-means) algorithm to optimize the topology of information transfer among deployed sensor nodes. Etiquette is created for multi-hop transmission, from which the information gathered from every neighboring node is sent to the technology for the external detecting connector, further balancing and reducing the system’s usage of energy. It evenly distributed energy usage across sensor nodes and greater average transmission power which leads to the energy gap issues. The SDCS developed by Han et al.^29^ uses an acoustic sensor network submerged beneath the water to collect data in strata. It's what separates the network's top layer from the deeper layers. The top layer affects the massive water velocity and the nodes move with water. In the deeper layer, the groundwater velocity is lower, and the nodes are thought to be largely immobile. It improves the lifespan of the delivery services and decreases the consumption of energy. Therefore, it can shorten the time taken for data gathering. Due to the eager energy production of the underwater sensor nodes, Yu et al.^30^ have demonstrated an enhanced energy optimization clustering algorithm (EOCA). The remaining power of each node that transports the underwater sensor network is isolated at the destination and the submarine sensor nodes. This builds a consistent association among the transmission power of each subsea member node using the curve parameter. It is efficient, feasible, and scalable, and the package ratio will be high. Moreover, the electricity supply is finite and requires more money to recharge or change (see^31–33^).

Based on the distance between sensor nodes and the destination nodes as well as the residual radiation of a normal node, Wan et al.^34^ have evaluated an energy-efficient adaptive clustering routing algorithm. It can prevent cluster heads from dying too soon apart from the base station caused by an increasingly competitive market zone that results in a greater energy load. It conserves system energy and increases the network’s operational lifespan. Thus, it is more difficult to cluster the sensor nodes from each data. Yan et al.^35^ suggested underwater cyber-physical system (UCPS) explores a challenge with energy-efficient data collecting. In the initial stage, the total weights are reduced with the improved network design, an updated routing alternative is offered for the base station to transmit the accumulated information to an information collector, hence extending the system reliability. The performance of the system can be extended by the architecture algorithm to solve. Hence, when several devices are combined it is more complicated. Chen et al.^36–38^ highlighted selective dynamic coded cooperation (S-DCC) as an excellent transient-coded collaboration approach. The cooperative nodes aggressively and brings the entire data transfer blocks, while the detector nodes preferentially accept and process these blocks in conformance with their immediate deciphering findings. The signal generator conveys the additional blocks during the processing period and send them to the participating nodes, while dynamic decoding may not actually be required. It is feasible and energy efficient. However, it is difficult to determine energy utilization.

Bouabdallah et al.^39^ describe a multi-channel Underwater Medium Access Control (MC-UWMAC) protocol establishing communication that is collision-free. A node can be provided with a high communication system by eliminating interference on each of the network control routes to the best of its ability by implementing a quick and effective mutual authentication process. It produces better results and is energy efficient. Thus, the missing receiver problem affects the network^40–42^. Khasawneh et al.^43^ developed a location-free Reliable and Energy-efficient Pressure-Based Routing (RE-PBR) sensor communication protocol. Location-free and location-based routing protocols exist. Sensor nodes calculate from source nodes using location. It broadcasts data packets with source node information. The routing protocol improves. However, the communication imbalance problem should be solved to improve the dimension system. Table 1 illustrates the reviewed works with their merits and demerits.

**Table 1:** Reviewed works with their merits and demerits.

Reference	Method/ Algorithm	Performance metrics	Limitations
Wang et al. ^26^	DC-K-means algorithm	Greater transmission power	Greater network demands result in an utility gap issue
Han et al. ^29^	SDCS	Increases lifespan and delivery services	There is a lack in improving the process of data
Yu et al. ^30^	EOCA	Efficient, feasible, and scalable	The replacement cost is more
Wan et al. ^34^	Adaptive clustering routing algorithm	Increases lifespan and energy	It is complicated method to cluster the sensor nodes
Yan et al. ^35^	an underwater cyber-physical system (UCPS)	efficient	Lack of combining several devices
Chen et al. ^36^	selective dynamic coded cooperation (S-DCC)	Efficient and feasible	Difficult to calculate energy consumption
Hou et al. ^40^	EULC algorithm	Efficient in energy usage, maintenance and lifespan	Lack in security
Khasawneh et al. ^43^	(RE-PBR	Improves performance	Lack in communication imbalance

## Background

This section deals with the system modeling of our proposed UWSN deployment in a dynamic nature. This provides a better idea about the sensors used in dynamic nature and encloses the variation between stationary and dynamic sensor nodes.

### Energy consumption modeling

This section employs the model of underwater energy consumption. The packets are received via node and the minimal power is $PW_{0}$. Where, $PW_{0} T(L)$ is the minimum power transmission and the attenuation function is $T(L)$. The following expression calculates both receiving and transmitting energy consumption^44–46^.

1 $$R_{eg} = N_{t} PW_{0}$$

2 $$T_{eg} (L) = N_{t} PW_{0} T(L)$$

The receiving energy consumption is $R_{eg}$ and the transmitting energy consumption is $T_{eg}$. The period taken to receive packets is $N_{t}$.

3 $$T(L) = b^{L} L^{1.5}$$

4 $$b = 10^{{{\raise0.7ex\hbox{${\beta (F_{c} )}$} \!\mathord{\left/ {\vphantom {{\beta (F_{c} )} {10}}}\right.\kern-0pt} \!\lower0.7ex\hbox{${10}$}}}}$$

5 $$\beta \left( {F_{c} } \right) = 0.11\frac{{F_{c}^{2} }}{{1 + F_{c}^{2} }} + 44\frac{{F_{c}^{2} }}{{F_{c}^{2} + 4100}} + 2.75 \times 10^{ - 4} F_{c}^{2} + 0.003$$

**Figure 1:**
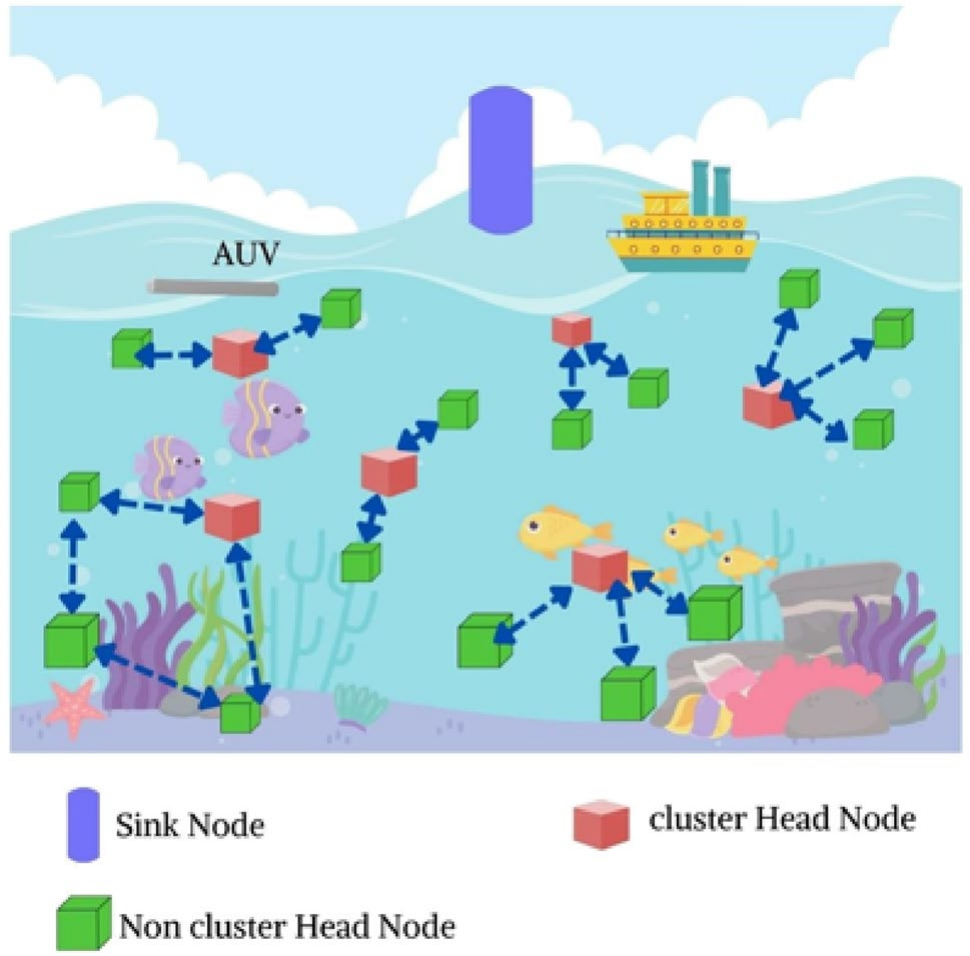
Proposed schematic for the proposed multi-hop routing approach in UWSN.

The frequency is $F_{c}$ and the absorption coefficient is $\beta \left( {F_{c} } \right)$. The spacebetween receiving and transmitting nodes is *L*.Fig. 1 depicts the schematic multihop model. Within the marine environment, the random distribution is under acoustic sensors^47–49^. After a cluster has formed, the nodes that make up the cluster and the cluster head are the only ones that can't be relocated. The sensor nodes are installed on the surface of the region under surveillance. The network model comprises a sensor node and the energy supplied and the destination node present in it. The unique IDs and equal initial energy present in an ordinary underwater node. The localization algorithms acquire the location of the node^50–52^. Distance from the Source node to the receiving node is used to regulate the flow of electricity. Cluster member nodes are broadcast to and collected from the head node with a single hop. When one cluster head node is physically closer to the sensor nodes than the others, data can be sent to them with a single hop.

### UWSN drift model

**Figure 2:**
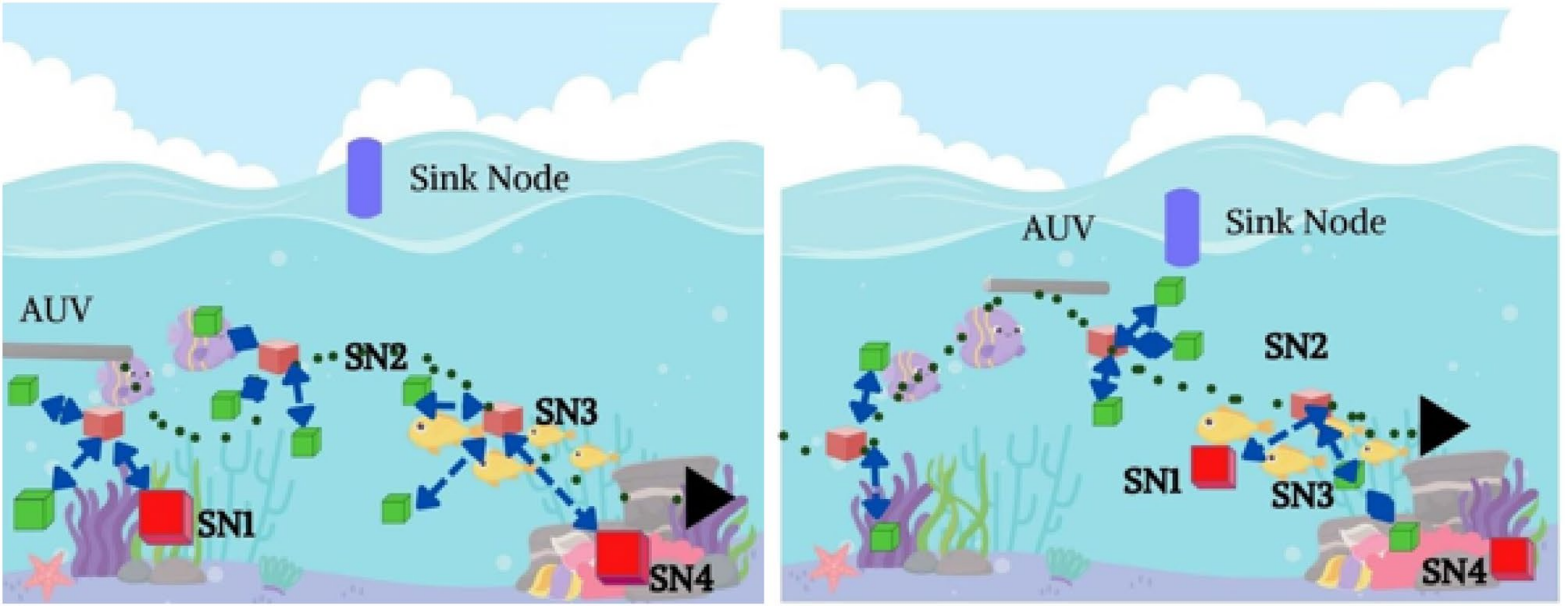
(**a**) Sensor nodes before the ocean current occurs, (**b**) After the drifting due to the ocean current.

The dynamic nature of underwater might have brought ocean currents and owing to this, the sensor node will change its position throughout the execution. The drift concept is outlined in Fig. 2a and b. To execute the ocean current we have considered a new concept known as Meandering Current Mobility (MC). Utilizing this concept, the trajectory of each sensor node can be evaluated. The motion of the underwater can be evaluated with the flow function $\varphi$.

Besides, the position of the underwater nodes is deemed as (a,b) and based on the rectangular coordinates it can be formulated as,

6 $$\hat{a} = - \frac{\partial \varphi (a,b,t)}{{\partial b}}$$

7 $$\hat{b} = - \frac{\partial \varphi (a,b,t)}{{\partial a}}$$

At t moments the location of nodes at the X and Y axes are denoted as $\hat{a}$ and $\hat{b}$ respectively. Based on these factors the function flow can be formulated as,

8 $$\varphi (a,b,t) = - \tanh \left[ {\frac{b - M(t)\sin k(a - ct)}{{\sqrt {1 + k^{2} M^{2} (t)\cos^{2} \left( {k(a - ct)} \right)} }}} \right]$$

The phase velocity and wave number are indicated as c and k respectively. The amplitude function of the Meander $M(t)$ can be formulated as,

9 $$M(t) = M_{0} + \varsigma \cos (\varepsilon t)$$

The mean width of the Meander is $M_{0}$ and is measured in km, the frequency and amplitude are given as $\varepsilon$ and $\varsigma$ respectively. the parameters of MCs are set as shown in Table 2.

**Table 2:** Parameter settings.

Parameter	Range
k	$2\Pi /7.5$
$\varsigma$	0.35
c	0.13
$M_{0}$	1.3 km
$\varepsilon$	0.45
_Maximum range_	14 km

## Proposed clustering routing algorithmic demonstration

**Figure 3:**
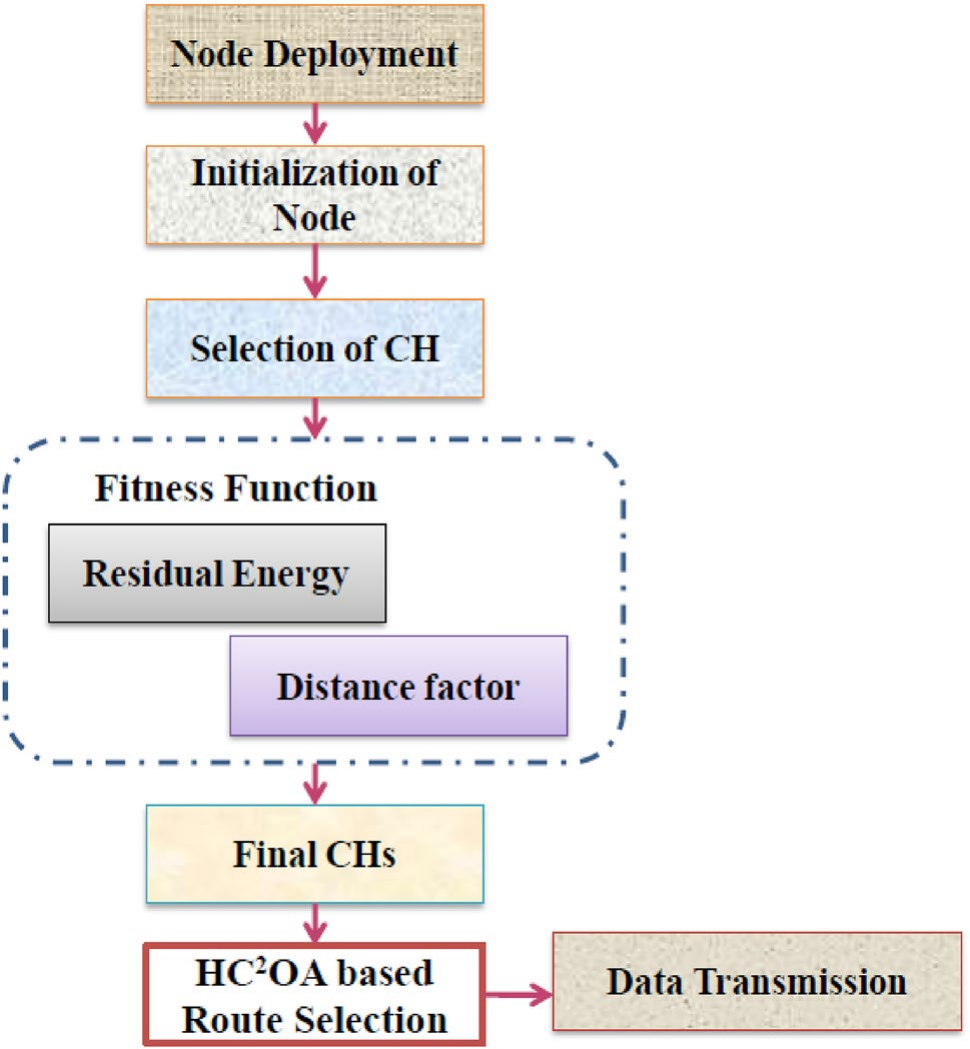
Proposed schematic for the multi-hop based route selection.

The proposed routing approach enclosed two stages namely (i) Selection of CH and (ii) Transmission of Data. From the system model, the network is split into various cubes and the selection of CH is effectuated for each cube which is deemed as a cluster. After the completion of selecting the CH, the other nodes become CMs. The CMs return the data t in a distinct hop which forwards the data multi-hops. The nodes between the routes of multiplehops are other selected CHs from the clusters and optimal routing is obtained with the proposed HC^2^OA as explained in Fig. 3.

### Selection of CH (cluster head)

For the efficiency of data underwater WSN CHs are very important by forwarding the information received from the CMs to the SN. Most of the protocols such as LEACH^53,54^ choose the CH without including the residual energy from all nodes that are involved. Residual energy must be higher to avert the early dying of nodes and therein significantly balances the efficiency of the network and energy. Thus it is necessary to deem the residual energy and if the node satisfies all the requirements to become a CH but its residual energy is lower than the average energy of the nodes in the clusters it will get quashed during the process. Moreover, we also included the distance factor while selecting the CH and the index used for the selection is framed below,

10 $$I_{a} = \frac{{\mu F_{aR} }}{{D.\,D_{A} }}$$

11 $$D_{A} = \frac{1}{M - 1}\sum\limits_{m = 1}^{M - 1} {D_{am} }$$

The residual energy possessed by node a is $F_{aR}$ and constant factor is $\mu$. The distance between node a and SN is implied as D. the mean distance between node a and other respective nodes in the specific cube is $D_{A}$. The total number of nodes in the cube is M and the distance between the node a and m inside the cube is $D_{am}$. From Eq. (6) it is observed that the node with more residual energy and a short distance to the SN has more possibility to be chosen as a CH than the other nodes in the cluster.

The value of $I_{a}$ each node inside the cube is estimated and forwarded to all the nodes of the cluster along with the ID and chosen the CH with the highest value $I_{a}$. The selected CH will broadcast the information to all CMs and each CM send an acknowledgment message to the CH and thus be accepted as CMs. Similarly, the CH forwards information such as the position, ID and the possessed residual energy to the SN via a packet.

### Transmission of data

Data is transmitted intra- and inter-cluster and the CH used Time Division Multiple Access (TDMA)^55^ to give CMs time slots to send information-carried packets to their respective CHs in a single hop. To save energy, CMs sleep after transmitting data. The CHs use multi-hop Carrier Sense Multiple Access with Collision Detection (CSMA/CD)^56^ to send CM packets to the SN. Thus, the optimal multi-hop paths must be chosen, which the proposed HC2OA approach can achieve.

#### Multi-hop routing using HC^2^OA

The optimal multi-hop routing in UWSN is achieved with the proposed HC^2^OA algorithm. The following section elucidates the CSO algorithm, CO algorithm, and hybridization and objective function for enabling optimal hops for the proposed multi-hop routing.The solution encoding will present the solution for the selection of hops in the proposed multi-hop routing. The hops are achieved with the chosen CHs and during the transmission; the routing enables and ensures minimum information loss. Moreover, with the reduced energy loss the delay that occurs during the effectuation of routing is also minimized. Meanwhile, the HC^2^OA algorithm is utilized for the selection routing between the source and destination in UWSN. Hybrid CSO is used for the selection of best possible routes for the communication of information from the CM to SN and viceversa. CSO is used for the selection of routes in the locally available and the multi-hops are made accordingly. For the global node selection, we have adopted the CO algorithm and or performed the multi-hops while transmitting the data from underwater. The natural behavior of the cat is utilized for the featuring of CSO which usually spends most of the time resting and the rest of the time finding prey and thus saving energy. The steps involved in the CSO^25^ are (i) Seeking mode and (ii) Tracing mode.

Seeking and tracing indicate resting and food-searching locations. The initialized cats are divided into two sets—the seeking mode set and the tracing mode set—in N-dimensional space. Few cats have a tracing mode. The mixture ratio (MR) must be lower to separate cats. In the UWSN the nodes that are not needed for the transmission are considered in sleeping mode and active modes are considered tracing mode. Meanwhile, the fitness functions are evaluated for each cat after settling into the respective groups. The best cat of all the cats is chosen and stored in the memory. These following steps are followed till it attains the stopping conditions.While resting the cats have awareness of what happens in their surroundings and based on the situation they transfer to the next hop node. For instance, there are some parameters that decide the position of the cat and are elucidated below,

- The copies are made with the Seeking Memory Pool (SMP) for the respective upcoming hop.
- Based on the old and new dimension values the Seeking Range for the selected Dimensions (SRD) is determined^26^.
- The counts of dimensions to be changed (CDC) are determined by varying the dimensions.
- Self Position Consideration (SPC) is used to define the existingformofthe Boolean Variable.

Apparently, the seeking mode process is explained below,

- If SPC = 1, the generation of SMP copies of cats is effectuated and evaluated the hop from the generated number of copies.
- Based on the copies of the cat the CDC is evaluated and the formula is defined as follows,
  12 $$Y_{n} = \left( {1 \pm SRD \times \eta } \right) \times Y_{p}$$

$Y_{n}$ is the next hop that cat follows and the current hop is $Y_{p}$ and arbitrary number $\eta$ and falls under the range of 0 to 1. If all cats have the same value after fitness function estimation, the cat is selected based on probability 1 and formulated as shown below,

13 $$\Pr ob_{i} = \frac{{\left| {E_{i} - E_{b} } \right|}}{{E_{\max } - E_{\min } }}$$

The $\Pr ob_{i}$ is the prospect of the existing cat with appropriateness value $E_{i}$, the maximum and minimum fitness value is $E_{\max }$ and $E_{\min }$ correspondingly. For minimum and maximum optimization issues it is set as, $E_{b} = E_{\max }$ and $E_{b} = E_{\min }$ respectively.The prey-searching process is explained in this mode. The next hop node and velocity at which the cat relies on the prey’s velocityis formulated using the following equation,

14 $$V_{i} = v_{i} + \eta_{1} \times r\left( {Y_{best} - Y_{i} } \right)$$

The predefined value is r = 2.05, the best node hop of the cat is implied as $Y_{best}$ and $Y_{i}$ is the current best cat hop. The formulation of cat hop is evaluated as,

15 $$Y_{new} = Y_{old} + v_{i}$$

The route of hop of the old and new location are represented as, $Y_{old}$ and $Y_{new}$ respectively.The selection of termination or halt condition is predominant to achieve the optimized solution and provide better convergence results. Some of the important termination criteria are iteration number and runtime of the process^57^.

### Cheetah optimization algorithm (CO)

For the global search of node CO^58^ is adopted and is based on the patrolling or scanning behavior of Cheetahs while effectuating the detection of prey. The attack of prey includes three steps (i) sit and watch, (ii) wait till the prey reaches closer, and (iii) attack. The transmission of data may be stopped for several reasons such as energy limits, breakdown, and so on. The numerical model of CO includes searching, sitting and waiting, and attacking and is elaborated in the following.

- *Searching:* the prey is found with the help of scanning around the search space or nearby.
- *Sitting and waiting:* The next step after finding prey is sitting and waiting for the right situation.
- *Attacking:* It includes two phases:

- *Rushing*: The Cheetah hops toward the next nodes with the maximum speed after finding the prey.
- *Capturing*: With the flexible speed Cheetah made the next hops to reach the node.

When there are so many nodes in the cluster then the Cheetah will scan and find the particular node to transfer the data. Meanwhile, if the nodes are scattered and active then the active mode will switch on, however, it consumes a little more energy^59^. Such the transmission mode chooses the mode based on the conditions, coverage area, and chain of the search space to transmit the data. It can be modeled numerically as,

16 $$Z_{n} = Z_{p} + \kappa .\vartheta$$

The new hop and current hop of the cheetah are denoted as $Z_{n}$ and $Z_{p}$ respectively. The step length of the cheetah during the multi-hop is $\vartheta$ and $\kappa$ is the random parameter of the system.While effectuating the penetratingform the node might have visible to the cheetah and make the hop-by-sit and-wait strategy. It can be framed as,

17 $$Z_{n} = Z_{p}$$

Here, $Z_{n}$ and $Z_{p}$ are the updated and present nodes of the cheetah and do not allow all the cheetahs to alter the node to avert an untimelyjunction. Cheetahs use two parameters to hop to the next node such as speed and flexibility^60–62^. After deciding to hop to the next node it will effectuate it at full speed. The attacking strategy of the cheetah is formulated as,

18 $$Z_{n} = Z_{B} + \kappa_{1} .\tau$$

Here, $Z_{B}$ implies the current node and the best position to transmit the data.

### Hybrid optimization algorithm: HC^2^OA

**Figure 4:**
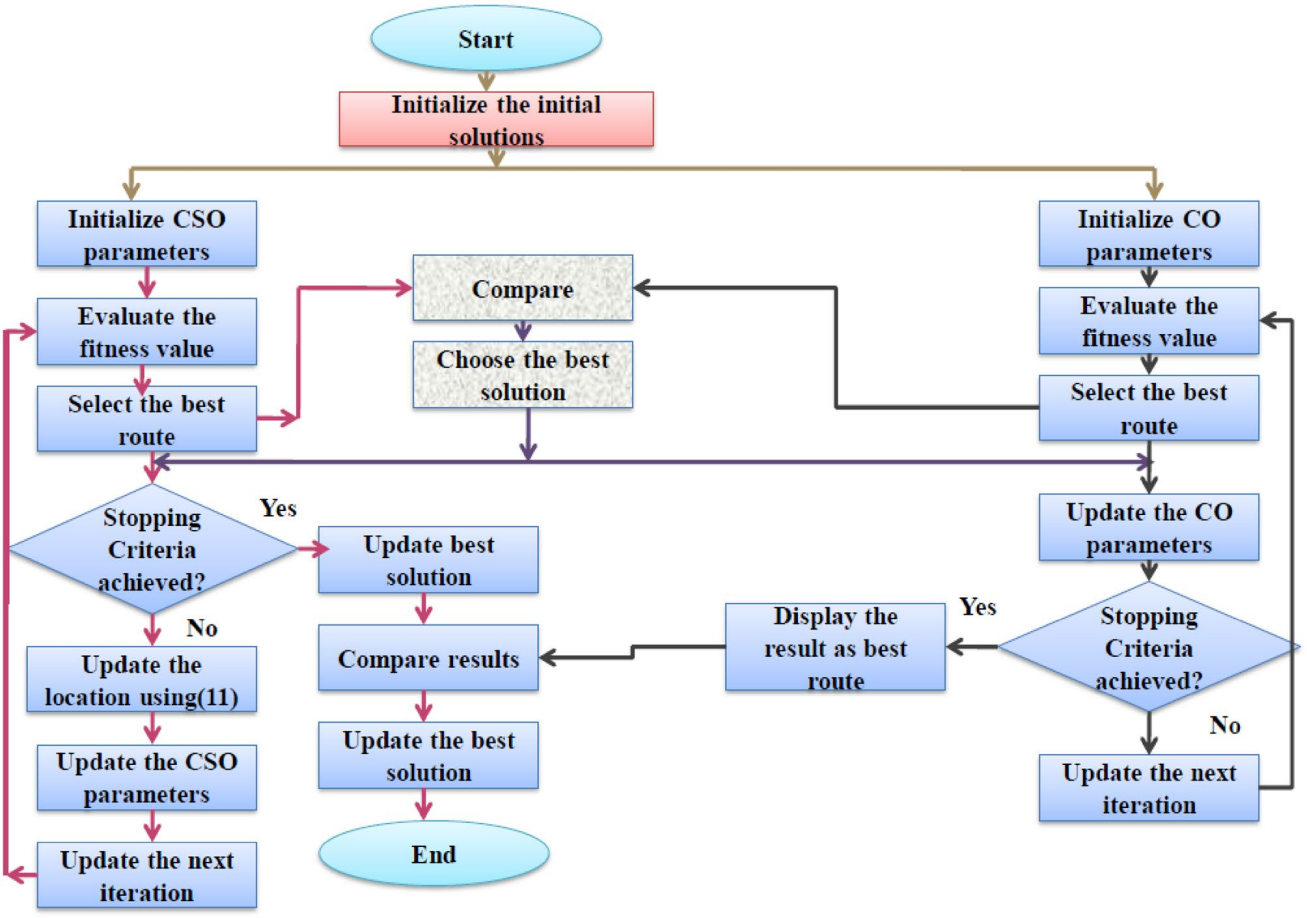
Proposed HC^2^OA approach for multi-hop routing in UWSN.

It is the modified version of CSO and CO and utilizes the merits of both algorithms and thus handles the exploitation and exploration issues. Moreover, it might have led to achieving the global optimal solution. It has one control parameter and thus updates the location hop of the data adaptively. The proposed HC^2^OA-based multi-hop routing is explained in Fig. 4.

The proposed HC^2^OA ensures the most advantageous routing to advance the information from the basis to the destination. Based on the optimal route the CH sends the IDs, residual energy, and position to the SN. In UWSN mostly the destination is SN and the CHs become the source node. Based on the selected optimal multi-hop route using the proposed HC^2^OA the information carried packets are passed to the SN. After completing the transmission of the first round of data the entire system will analyze to verify the lingering power of the CHs. If the energy is higher than the same CH is maintained for the next round also, otherwise, a new CH will select based on the procedures explained in Sect. "Selection Of Ch (Cluster Head)". The process will repeat until it reaches the termination criteria. The pseudo-code used for the proposed multi-hop data transmission is displayed in Algorithm 1.
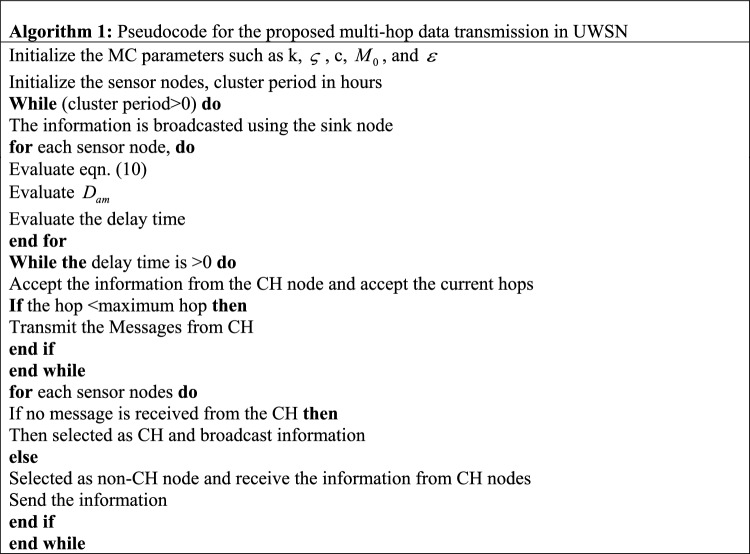


#### Computational complexity

The HC^2^OA computational complexity is based on the maximum repetitions (*T*), dimensions (*D*) and the number of cat cheetah solutions (*Y*). In the worst case, each iteration based on the computational complexity of the sorting process is $O(T \times m^{2} )$. Where, $O(m)$ is the initialization process computational complexity and $O(T \times m \times D)$ is the all-search agent position updating step. The number of cats or cheetahs is *m* and $O(m^{2} )$ is the local optimum to find a time complexity. Here, $O\left( {T \times m^{2} \left( {m^{2} + m \times V} \right)} \right)$ and $T$ are the number of variables and a maximum number of iterations.

## Result and discussion

**Figure 5:**
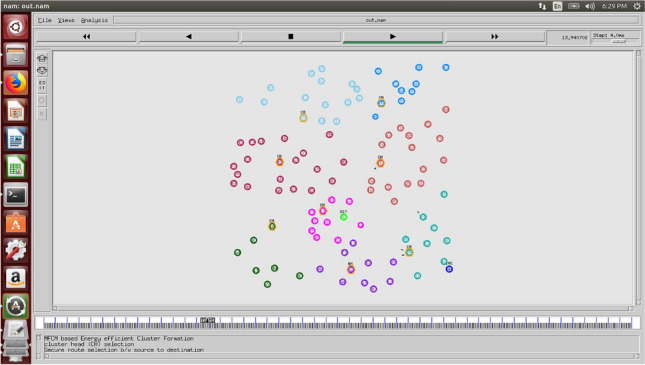
UWSN sensor node distribution in NS2 simulator.

This section encloses the experimental setup, parameter explanation, performance analysis of our approach and comparative study in a wider context.The NS2 simulator and the sensor nodes are arbitrarily placed in the region of 5500 m × 5500 m × 1500 m and the SN has the coordinate of 3000 m × 3000 m × 0. The system is split into cubes and is 64 cubes. The sensor nodes are taken fall under the range of 300 to 600 for various setups. The transmission of data is in the range of 2048 bps and the length of the packet is 1024 bits. This measures data transmission time. Broadcasting and message packets are 64 bits and sound travels 1500 m/s. Table 3 shows the proposed work parameters. The sensor node distribution in the NS2 simulator is shown in Fig. 5.

**Table 3:** The simulation parameters.

Parameters	Ranges
Simulator	NS2
Number of underwater sensor nodes deployed	300 to 900
Area	5500 m × 5500 m × 1500 m
Power consumption at the destination	_0.157 W_
Power consumption at the source	_50 W_
Time is taken for simulation	_2500 rounds_
Range of transmission	_40 m_
Traffic method	_CBR_
Initial energy	_0.5 J_
Maximum retransmission	_1_
Depth at which nodes are deployed	_25 m_
Wind speed	_16 m/s_
Mode of modulation	_OFDM_

### Outcomes based on the comparative study of state-of-art works

**Figure 6:**
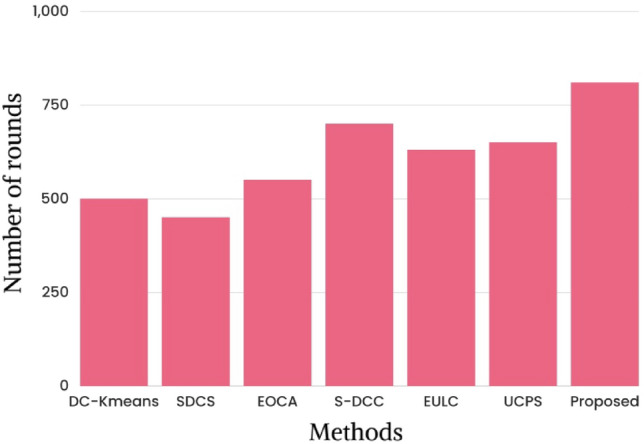
State of art study based on the initial node dead.

In order to assess the durability of the network, this research uses a small set of performance metrics, including final node dead, half node dead, and initial node dead. The state-of-the-art research based on an initial node death is shown in Fig. 6 in terms of rounds. The state-of-art methods like DC-K-means^26^, SDCS^29^, EOCA^30^, S-DCC^36^, EULC, UCPS and proposed framework take place of an initial node in the round of 500th, 450th, 550th, 700th, 630th, 650th and 810th correspondingly. Due to prolonging the network lifetime, this investigation offers that the proposed method outperformed the superior assessment of other methods.

**Figure 7:**
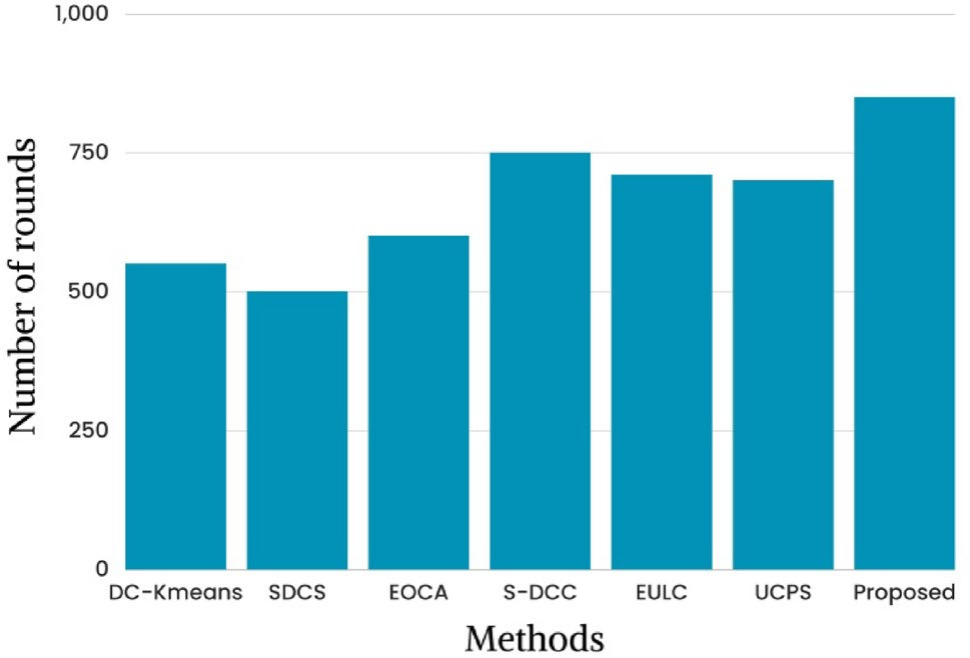
State of art study based on the half-node dead.

Figure 7 shows the latest half-node dead study based on rounds. During cluster head node selection, low-energy nodes are prevented and network nodes are equally distributed. The framework finds the most energy-efficient paths between sensor nodes and cluster head nodes. DC-K-means^26^, SDCS^29^, EOCA^30^, S-DCC^36^, EULC, UCPS and the proposed framework show dead half nodes in the 550th, 500th, 600th, 750th, 710th, 700th, and 850th rounds. Because of the longer network lifetime, this investigation suggests that the proposed method outperformed good results in other methods in terms of evaluation without believing the node residual energy, the cluster head node is selected randomly in which the existing methods offer worst performances than the proposed.

**Figure 8:**
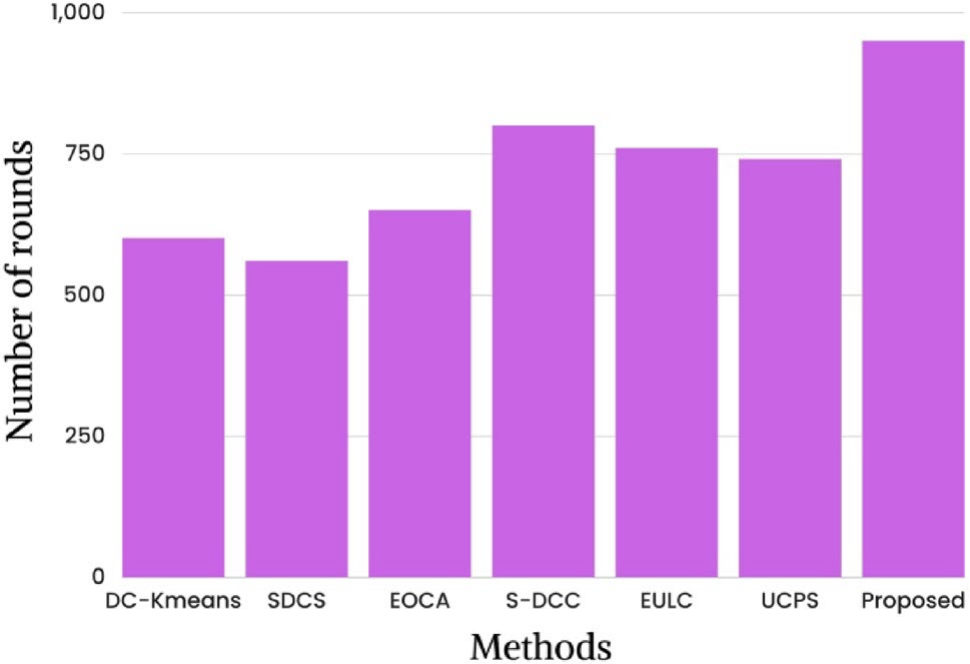
State of art study based on the final node dead.

Figure 8 displays the current state-of-the-art research regarding the number of rounds based on the final node dead. The proposed framework, along with other modern methods like DC-K-means^26^, SDCS^29^, EOCA^30^, S-DCC^36^, EULC, UCPS, and proposed work replace the terminal node in the 600th, 560th, 650th, 800th, 760th, 740th, and 950th iterations, respectively. This research proposes a hybrid optimization based on residual energy to determine which nodes in a cluster should be made the leaders. Based on the results of this study, it appears that the proposed method's superior network lifetime makes it the clear winner in terms of evaluation.

**Figure 9:**
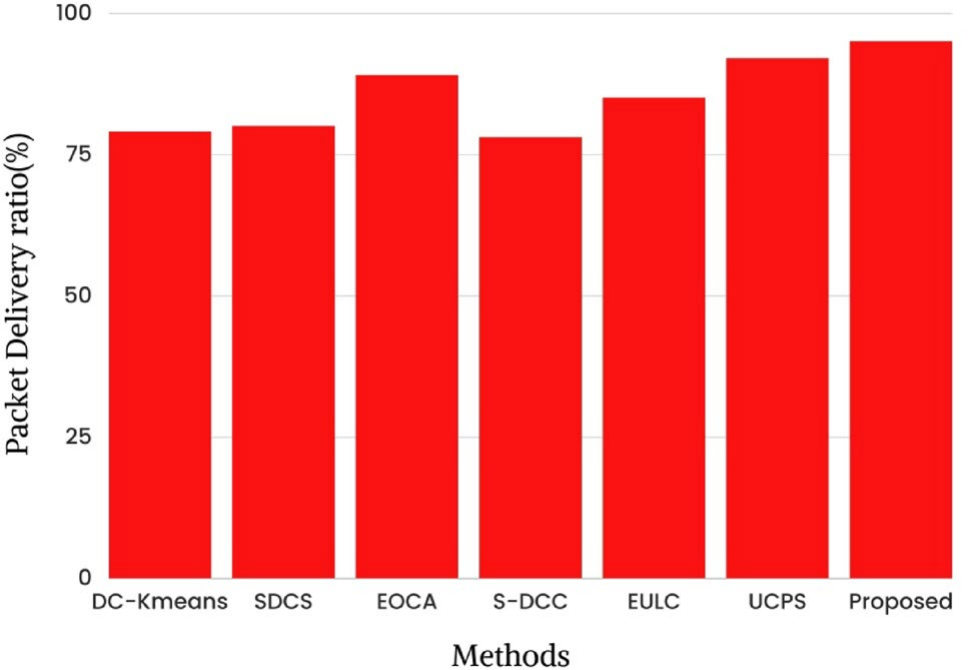
State of art study of packet delivery ratio.

Figure 9 depicts the most up-to-date research on the packet delivery ratio in relation to the total number of rounds. It has been estimated that 79%, 80%, 89%, 78%, 85%, 92% and 95% of packets are delivered successfully using state-of-the-art methods like DC-K-means^26^, SDCS^29^, EOCA^30^, S-DCC^36^, EULC, UCPS, and the proposed work. This research showed that the proposed method had a better packet delivery ratio than previous research. The result is displayed in Table 4.

**Table 4:** Comparative study based on packet-delivery ratio.

Methods	Packet delivery ratio (%)
DC-Kmeans	79%
SDCS	80%
EOCA	89.67%
S-DCC	78.2%
EULC	85%
UCPS	92%
Proposed	95%

**Figure 10:**
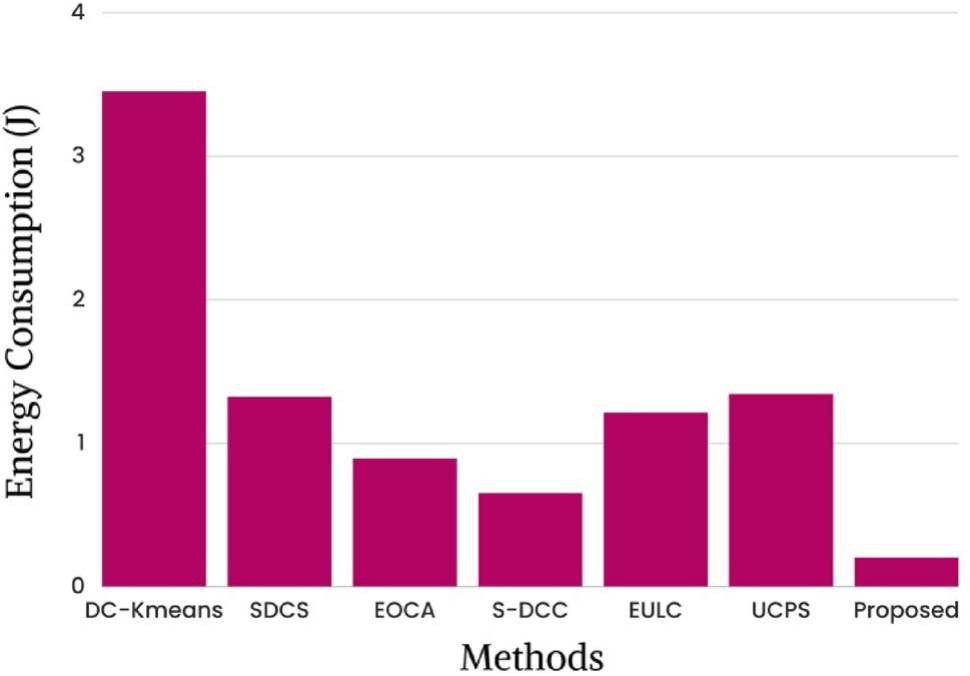
State-of-the-art study of packet delivery ratio with respect to energy consumption.

Current research on the optimal packet delivery ratio versus energy usage is depicted in Fig. 10. By using the proposed framework, the most efficient routes between sensor nodes and cluster head nodes can be determined. Energy consumption of 3.45 J, 1.32 J, 0.89 J, 0.65 J, 1.21 J, 1.34 J and 0.2 J are shown for state-of-the-art methods like DC-K-means^26^, SDCS^29^, EOCA^30^, S-DCC^36^, EULC, UCPS, and proposed work respectively. The results are displayed in Table 5.

**Table 5:** Results based on energy consumption.

Methods	Energy consumption (J)
DC-Kmeans	3.45
SDCS	1.32
EOCA	0.89
S-DCC	0.65
EULC	1.21
UCPS	1.34
Proposed	0.02

### Outcomes based on network coverage area influences

**Figure 11:**
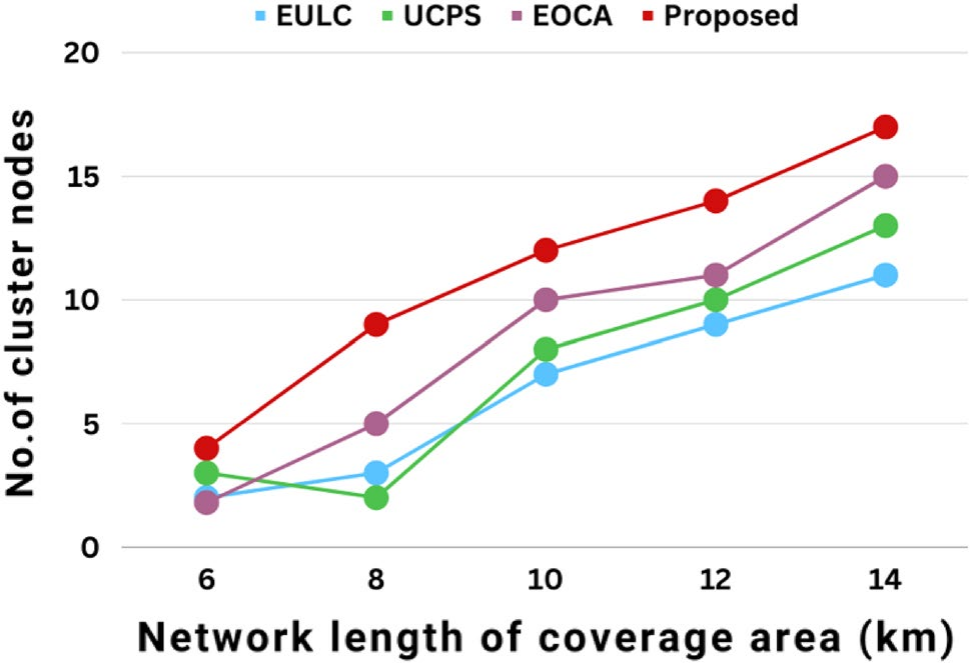
The variation of cluster head nodes in relation to the network coverage area performances.

The transmission range area with the variability of the CH node is plotted in Fig. 11 when different clustering methodologies are used. The X-axis represents the transmission range area edge length. Whilst also expanding the multi-hop coverage area network, progressively boost the amount of CH nodes using strategies like UCPS, EULC, EOCA, and the proposed scheme. The suggested technique made use of a larger network coverage area. Boost the ranges among each node as the density of underwater sensor nodes reduces.

**Figure 12:**
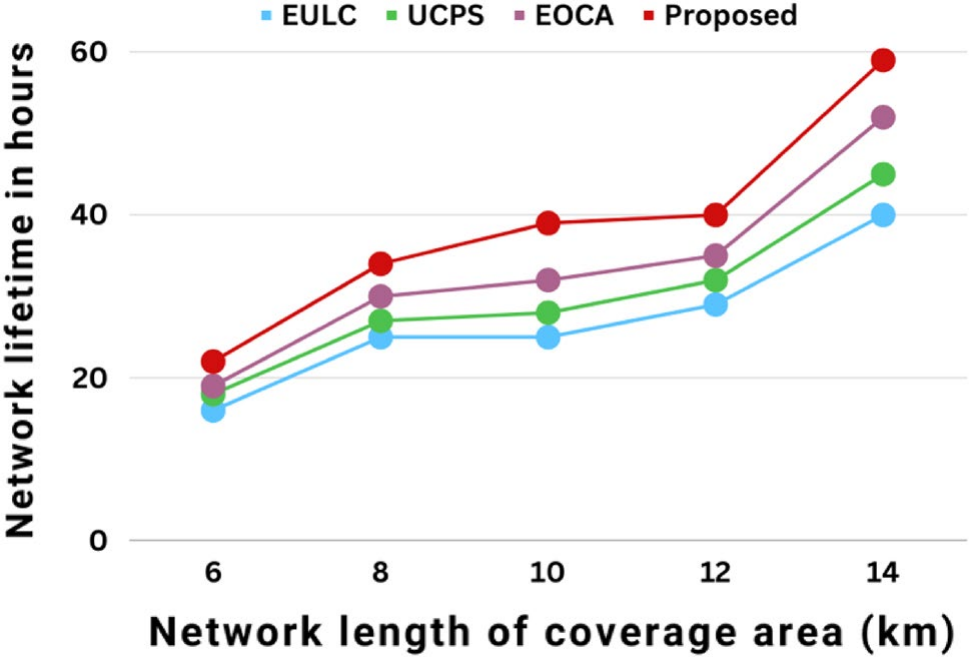
The multi-hop network lifetime in relation to the network coverage area performances.

Figure 12 depicts the multi-hop network lifetime based on network coverage area. The energy usage for each package communicates the message as the connectivity coverage area expands. The current technique regulates the rate of energy usage for each UWSN. The proposed method outperformed existing techniques such as UCPS, EULC, and EOCA in terms of lifetime effectiveness.The network lifetime of proposed work increases with the coverage area and when the coverage area is around 14 km the average network lifetime is around 60 h as shown in Fig. 12.

**Figure 13:**
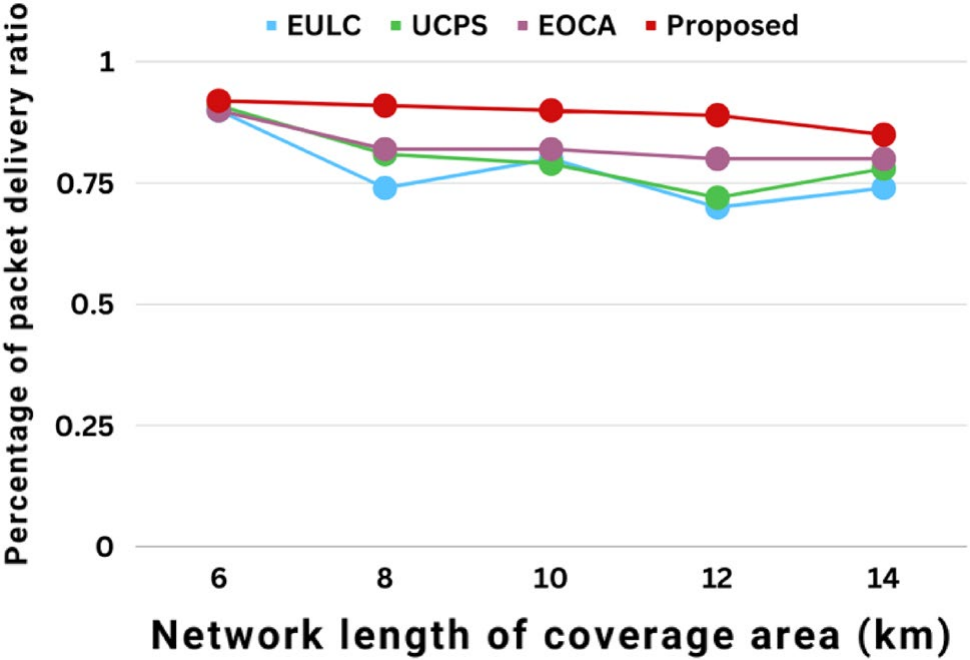
The package delivery ratio in relation to the network coverage area performances.

Figure 13 depicts the network coverage area by package delivery ratio. The connectivity coverage area expands as the package delivery ratio is increased using diverse methods such as UCPS, EULC, EOCA, and proposed methods. At first, the suggested technique is slow, even though the coverage area is increased. Choose the right CH nodes to ensure a higher package delivery ratio. When contrasted with current schemes, the proposed method exceeded better outcomes by improving the efficacy of the package delivery ratio.

## Conclusion

In order to select the best multi-hop path from the CH to the SN, this research introduces a brand-new Hybrid Cat Cheetah optimization algorithm (HC2OA). There are many clusters in the network, each with its own CH and many members (CM). Maximize the benefits by picking the CH that has the least amount of residual energy and the shortest travel time. In order to get from CH to SN, the proposed HC2OA takes into account multiple possible routes and chooses the optimal one. The NS2 simulation software is used to run simulations, and the outcomes are analyzed. The study found that the proposed design had significantly better results than the state-of-the-art works in terms of energy consumption, packet delivery ratio, and network lifetime. This study provides evidence that the proposed method outperformed the superior assessment of other methods by increasing the network's lifetime. When compared to other state-of-the-art methods such as DC-K-means, SDCS, EOCA, and S-DCC, the proposed method consumes 0.2 J less energy and achieves a 95% packet delivery ratio.

## Data Availability

All the data are available in the MS.
